# Theoretical Insights Reveal Novel Motions in Csk’s SH3 Domain That Control Kinase Activation

**DOI:** 10.1371/journal.pone.0127724

**Published:** 2015-06-01

**Authors:** Sulyman Barkho, Levi C. T. Pierce, Sheng Li, Joseph A. Adams, Patricia A. Jennings

**Affiliations:** 1 Department of Chemistry and Biochemistry, University of California San Diego, La Jolla, California, United States of America; 2 Department of Medicine, University of California San Diego, La Jolla, California, United States of America; 3 Department of Pharmacology, University of California San Diego, La Jolla, California, United States of America; Hungarian Academy of Sciences, HUNGARY

## Abstract

The Src family of tyrosine kinases (SFKs) regulate numerous aspects of cell growth and differentiation and are under the principal control of the C-terminal Src Kinase (Csk). Although Csk and SFKs share conserved kinase, SH2 and SH3 domains, they differ considerably in three-dimensional structure, regulatory mechanism, and the intrinsic kinase activities. Although the SH2 and SH3 domains are known to up- or down-regulate tyrosine kinase function, little is known about the global motions in the full-length kinase that govern these catalytic variations. We use a combination of accelerated Molecular Dynamics (aMD) simulations and experimental methods to provide a new view of functional motions in the Csk scaffold. These computational studies suggest that high frequency vibrations in the SH2 domain are coupled through the N-terminal lobe of the kinase domain to motions in the SH3 domain. The effects of these reflexive movements on the kinase domain can be viewed using both Deuterium Exchange Mass Spectrometry (DXMS) and steady-state kinetic methods. Removal of several contacts, including a crystallographically unobserved N-terminal segment, between the SH3 and kinase domains short-circuit these coupled motions leading to reduced catalytic efficiency and stability of N-lobe motifs within the kinase domain. The data expands the model of Csk’s activation whereby separate domains productively interact with two diametrically opposed surfaces of the kinase domain. Such reversible transitions may organize the active structure of the tyrosine kinase domain of Csk.

## Introduction

The Src family of tyrosine kinases (SFKs) constitutes a central hub for cellular signaling and regulation. These kinases phosphorylate many protein substrates associated with cell growth, differentiation, adhesion, motility and invasion [1–4]. Thus, SFKs are proto-oncogenic enzymes that are tightly regulated through molecular and spatio-temporal mechanisms and are only transiently activated upon stimulation. All SFKs contain a conserved C-terminal tyrosine kinase domain composed of an N-lobe important for nucleotide binding and a C-lobe important for protein substrate recognition. SFKs also contain two N-terminal Src homology domains (SH2 and SH3) that down-regulate kinase domain activity through intramolecular contacts [5]. In the case of the prototypical SFK c-Src, crystallographic studies show that the SH2 domain recognizes a phosphotyrosine (pTyr-527) on the C-terminal tail of the kinase domain enforcing close contacts between the SH2 and kinase domains that inhibit catalytic activity. This domain-domain interaction is further stabilized by contacts between the SH3 domain and a polyproline linker connecting the SH2 and kinase domains. Interestingly, dephosphorylation of the C-tail by a protein phosphatase and/or interaction of the SH2 domain with a cellular adapter protein disconnects these inhibitory contacts and activates c-Src.

The C-terminal Src Kinase (Csk) serves as the master regulator and suppressor of all SFKs whose mis-regulation leads canonically to tumorigenesis [6]. Csk phosphorylates the C-tail Tyr-527 of c-Src inducing extensive structural rearrangements that result in enzyme down-regulation [7,8]. While Csk also possesses modular SH domains, notable differences between SFKs and Csk are apparent and most likely linked to regulation. First, whereas the inactive form of c-Src positions the SH3 domain on the N-lobe and the SH2 domain on the C-lobe of the kinase domain, in Csk both regulatory domains are positioned on opposite faces of the same N-lobe. Second, whereas the kinase domain of SFKs require phosphorylation of a tyrosine in a loop segment in the kinase domain (activation loop) for high activity, Csk lacks this tyrosine and does not require an activating phosphorylation step. Third, the architecture of the N-lobe is thought to render the kinase domain of c-Src intrinsically active whereas Csk is intrinsically inactive in the absence of SH domain interactions. Detailed mutagenesis studies indicate that the high catalytic activity of the Src kinase domain compared to Csk may be due to a network of interactions in the N-lobe that has the β4–5 loop as a hub [9]. While this loop activates the N-lobe in Src by interacting with nearby Arg-264 and the C-loop, these critical interactions are broken in Csk in place of new contacts between the β4–5 loop and a helix in the SH2 domain. In accordance with these novel interactions, removal of the SH2 domain significantly reduces Csk catalytic activity whereas the same deletion activates c-Src [10–12].

Despite the available data on Csk’s SH2 domain, its conformational heterogeneity and its effects on kinase function [13–15], motions in other parts of the molecule have been harder to link to functional regulation. The SH2 domain is central to Csk’s direct activation via the N-lobe [14,16]. A number of phosphotyrosine ligands including the Csk binding protein (PAG/CBP) [17], pragmin and bacterial peptide effectors possessing the EPIYA sequence [18] can bind to the SH2 domain and enhance catalysis. On the other hand, few studies attempted to address the role of the SH3 domain in the direct activation of Csk. Notably, a previous NMR study on the isolated SH3 domain identified potential interaction segments with the kinase domain by monitoring dynamic parameters upon complexation of a phosphatase peptide-bound SH3 domain with the isolated kinase domain [19]. Nonetheless, a clear view of domain motions, non-canonical interaction sites, and mechanism of activation in the intact kinase remained ambiguous. For example, while prior studies have shown that deletion of the SH2 domain in Csk lowers catalytic activity (i.e.-k_cat_/K_m_ for Src) by 30-fold, removal of the SH3 domain lowers activity by 11-fold [20]. These similar changes in activity upon SH2/3 domain removal suggest that the activation mechanism in Csk extends beyond simple SH2-kinase domain contacts and include both regulatory domains. Furthermore, removal of both SH2 and SH3 domains do not significantly lower kinase activity beyond the individual domains [12,20]. Such kinetic findings hint to a cooperative interplay between the SH2 and SH3 domains with regard to their ability to activate the C-terminal tyrosine kinase domain in Csk.

Our starting computational approach here provides new insights into the Csk scaffold and leads to the hypothesis that the SH3 domain may be involved in functional motions that are not readily apparent when examining static structures of the full protein. This newly-discovered flexibility is closely linked to established motions by the other regulatory regions like the SH2 domain. Indeed, our combinatorial approach allowed us to identify previously hidden motions in an important part of Csk that assists in directly activating the kinase. We discovered using aMD calculations that the SH3 domain is closely linked to the motion of the SH2 domain on the other end of the molecule; the engagement of one causes the other domain to disengage the kinase domain hinting to a possible cyclical, direct activation by the two regulatory modules. These simulations identify several types of SH3-kinase domain contacts, one of which not apparent in the X-ray structure, that are critical for activating the kinase domain of Csk. The effects of SH2/3 domain motions are corroborated by DXMS methods and linked to changes in the N-lobe of the kinase domain most notably the network hub, β4–5 loop. Moreover, our experimental interrogations of possible contacts between the SH3 and kinase domain show that a structurally intact, conserved SH3 domain is required for such efficient activation. These theoretical and experimental insights paint an image of Csk in which concerted motions in the SH2 and SH3 domains reciprocally communicate through the kinase domain. These transitions may be vital for complete catalytic activation of the central tyrosine kinase domain in Csk.

## Results

### Accelerated Molecular Dynamics (aMD) Simulations Sample Unique and Concerted Motions between CSK's Regulatory and Kinase Domains

Csk's X-ray structure [13] reveals that the modular SH2 domain can sample two distinct conformations in the absence of significant global changes in the rest of the protein. However, we have shown previously using Small-angle X-ray scattering (SAXS) measurements that Csk samples an ensemble of conformations in solution that may be relevant for kinase function [21]. To probe possible functional motions, we employed advanced molecular dynamics techniques [22] to access broader samplings of the conformational landscape in Csk. While this method initially applied to relatively small systems, aMD has also been used to examine large biomolecules such as Trypanosoma cruzi (T.cruzi) proline racemase and Get3, a component of the guided entry of tail-anchored protein pathways, and GPCR signaling [23–25]. The Get3 study is especially interesting in that it utilized aMD to define previously unexplored phase space and seeded rigorous calculations of free energy profiles of a large, multi-liganded complex.

**Fig 1:**
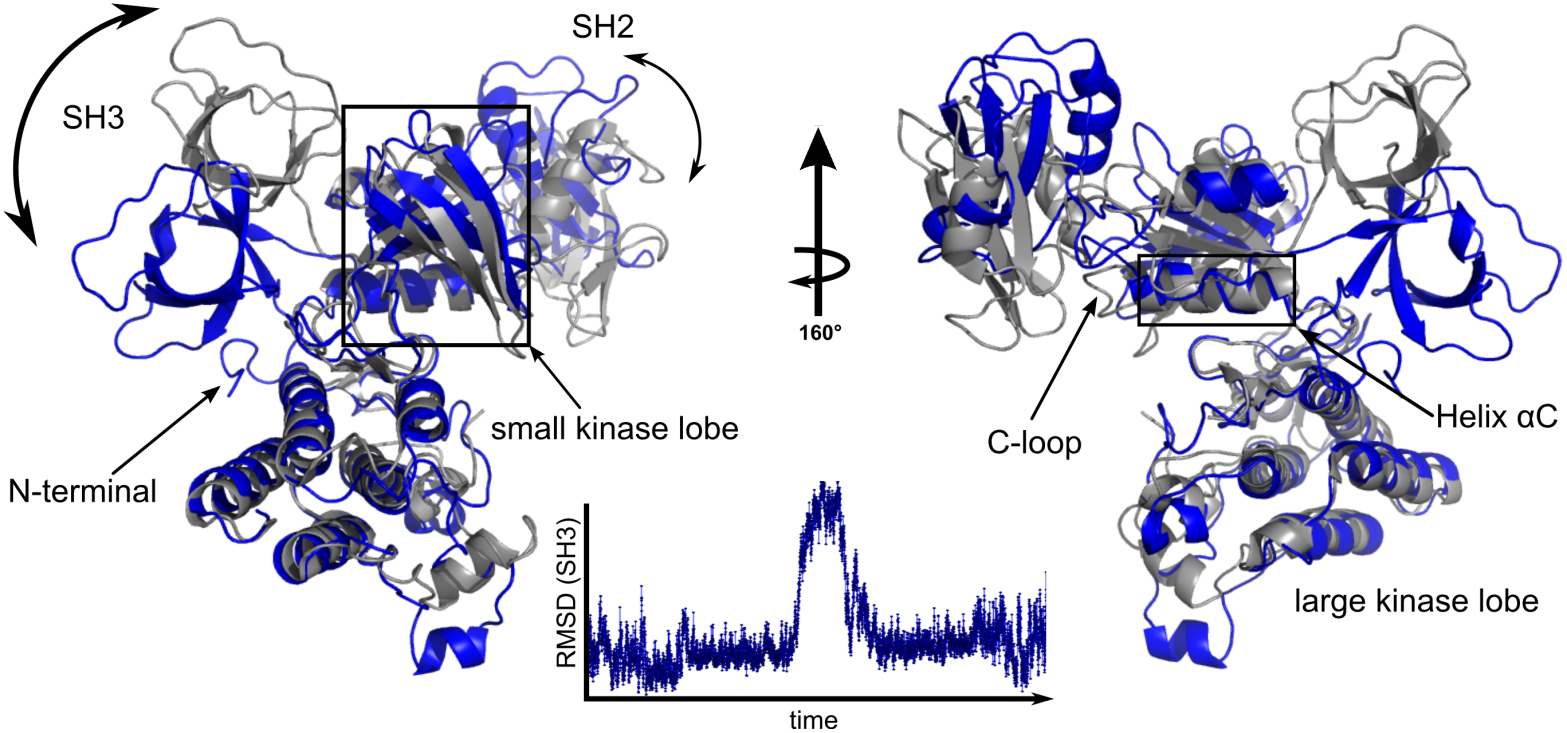
Csk’s global domain motions observed in aMD. Structural alignment of two representative frames from the aMD trajectory highlighting concerted transitions of the modular SH3 and SH2 domains of full-length apo Csk. The starting conformation (gray) is extracted from the crystal structure (PDB: 1K9A). A plot of root mean square deviation (RMSD) of the SH3 domain (1–70) as a function of aMD simulation time (200 ns).

A striking result from the aMD simulations of Csk is the observation of concerted and seemingly coupled global motions of the two noncatalytic domains (Fig 1). The trajectory shows an SH2 domain that disengages and moves upwards from the C-loop of the N-lobe, a site of canonical activation via the SH2 module [14,16]. Concurrently, the SH3 domain populates a new conformation by moving downward and engaging the kinase domain on the other side of the molecule before returning to its baseline coordinates (Fig 1). Although this reversible transition is the largest observed, a closer inspection of RMSD values for the SH2 and SH3 domains shows coordination of smaller motions about the kinase core, albeit with higher frequency for the SH2 (S1 Fig).

Although both domains are mobile, we chose to focus on the SH3 domain owing to the greater shift in position in our aMD simulations and the dearth of information on this domain in regulating catalysis (S1 Fig). Importantly, the large transition observed for the SH3 domain in the long simulation is also observed in a shorter, independent run that uses the same initial conformation of Csk (S2 Fig). An inspection of the SH3 domain docking surface on the kinase domain reveals significant polar/hydrogen bonding through side-chain interactions between the two domains. We performed contact analysis for every residue in the SH3 domain (residues 1–70) and the rest of the protein (residues 71–450) to interrogate functionally relevant interactions by calculating the “percent occupancy” for each inter-domain contact. Occupancy was defined if conditions of having a donor-acceptor distance of 3.8 Angstroms and an angle less than 35 degrees were fulfilled.

**Fig 2:**
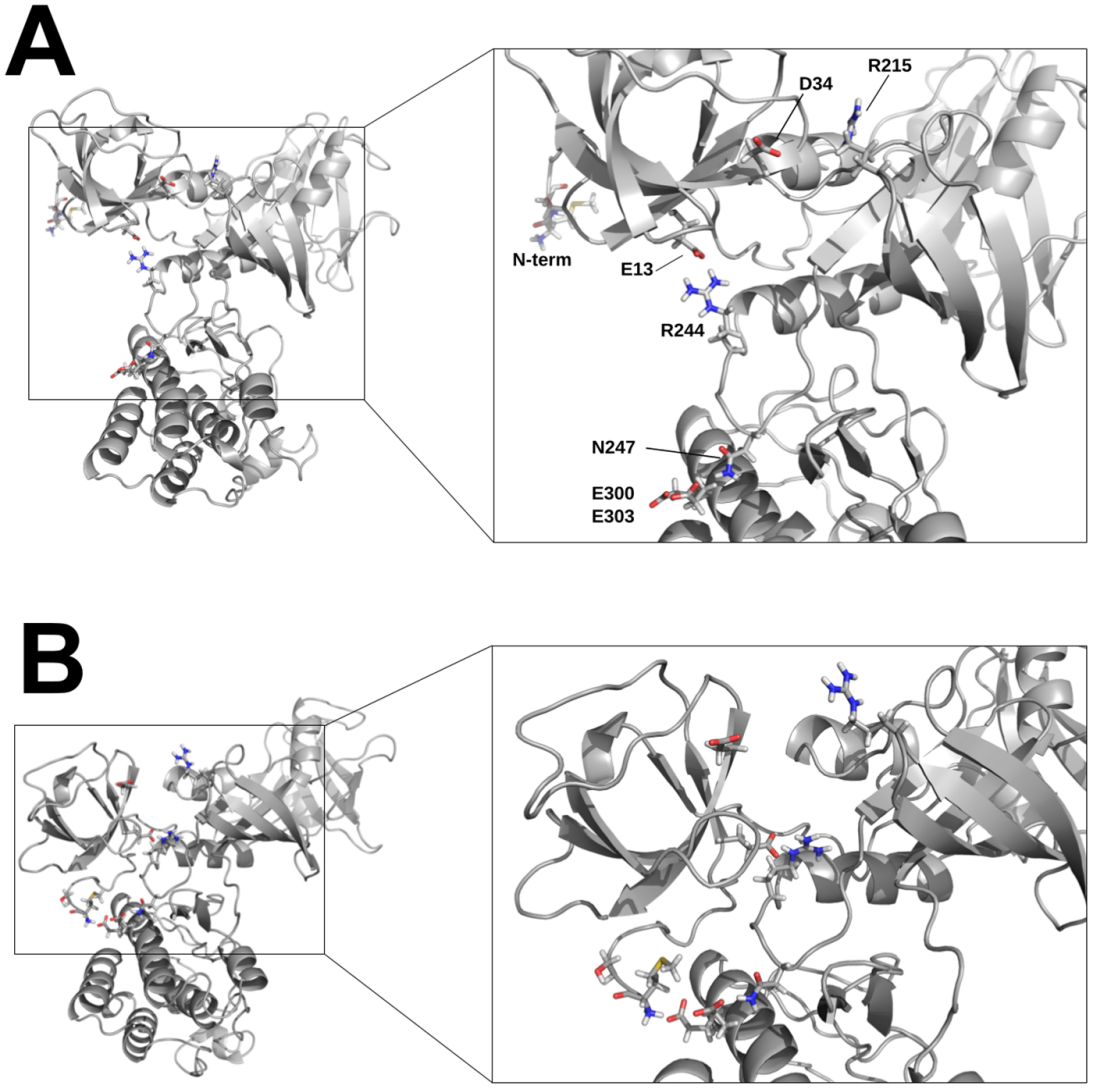
Functional interdomain contacts suggest SH3-mediated activation. High occupancy polar contacts at the SH3-kinase core interface in Csk show a flexible hydrogen bonding network in the starting, “SH3 up” state, A. The same network of residues is shown from a simulation frame of the “SH3 down” conformation, B.

In the X-ray structure of Csk, a central interaction between the SH3 and kinase domains is mediated by the charge pair E13-R244. Interestingly, trajectory inspection and contact occupancy analysis (S3 Fig) indicate that this contact is maintained throughout the long simulation despite being the most flexible since the R244 side chain undergoes significant rearrangement without severing contacts with E13 (Fig 2). Additionally, D34 occupies a conserved acidic position at the end of the RT loop in the SH3 domain. Occupancy analysis shows that D34’s acceptor residue on the kinase domain is R215, which is anchored in the β2-β3 loop of the N-lobe. Both E13 and D34 maintain high contact occupancy (~80% of simulation time) throughout the aMD trajectory.

A surprising result from the aMD simulation is that Csk’s N-terminal residues, which are part of a flexible segment not observed in the X-ray structure, participate in electrostatic inter-domain interactions and serve as a third contact region between the SH3 and kinase domains. Unlike E13 and D34, the acceptor sites in this interaction are predominantly two solvent-exposed glutamate side chains (E300/E303) on helix E of the large kinase lobe (Fig 2). This interaction could facilitate and/or stabilize the transient coupling interface between the two domains. Interestingly, an “N-tail” interaction was hinted to in a previous study [19] in the form of line broadening of the N-terminal residues in the NMR spectra for the isolated SH3 domain upon complexation with a phosphatase peptide and the isolated kinase domain; however, the corresponding kinase contacts were never identified.

### Interfacial Domain Contact Disruptions Have Differential Effects on Catalytic Function

**Fig 3:**
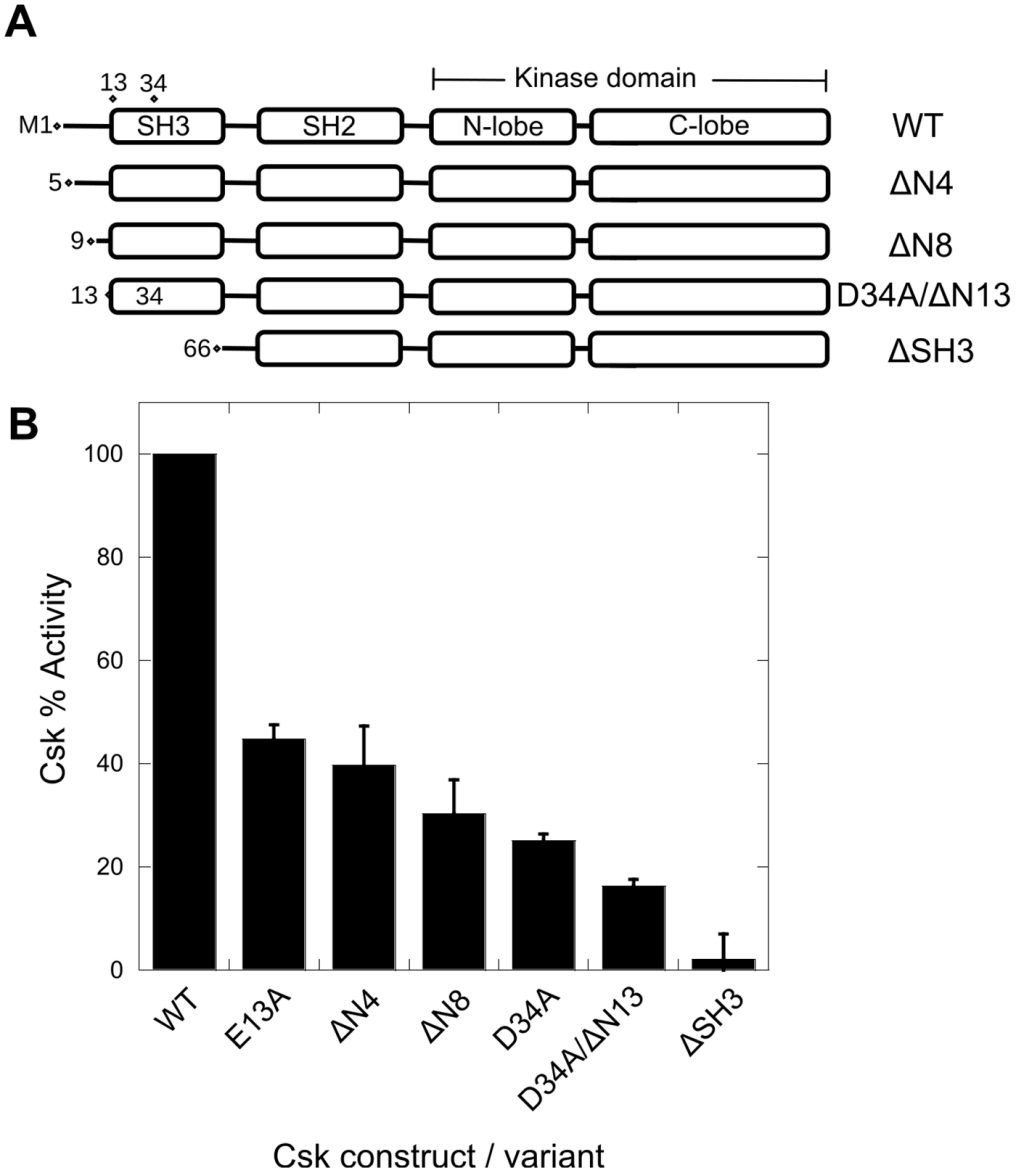
Perturbations to the SH3 domain contacts inhibit kinase activation. A) Schematic representations of some of the Csk constructs used in this study. B) The Csk kinase activity assay is used to determine the extent of SH3-mediated kinase activation. Kinase activity was monitored in a [γ-^32^P]ATP coupled radioactive assay in which a kinase dead substrate (kdSrc) is phosphorylated as a function of time. The reactions typically included 100 nM Csk, 5 μM kdSrc, 10 mM MgCl_2_, 1 mM MnCl_2_ and were initiated with 100 μM ATP at 23°C.

To test our aMD results, we performed experimental studies and initially generated a Csk construct that deletes the entire SH3 domain (ΔSH3; residues 66–450). For ΔSH3, we chose to retain the long, unstructured SH3-SH2 linker as previous studies deemed these residues to be essential for Csk’s activation and packing [14,19]. We found that ΔSH3 exhibited a ~ 33-fold reduction in catalytic activity towards the protein substrate kdSrc (Fig 3). These findings are similar to those previously reported by the Sun lab [20].

Next, we used alanine substitution to remove several charged contacts that were identified in the aMD calculations. The two acidic residues E13 and D34 maintain extensive occupancy with R244 and R215 of the small lobe, respectively, throughout the simulation (~80%) and, thus, are predicted to be important for regulating SH3 domain motion in Csk. The E13A and D34A Csk variants exhibited 60–80% reductions in Csk’s kinase activity towards kdSrc compared to the wild type enzyme (Fig 3). These findings highlight the significance of these charged residues with regard to kinase domain function.

To determine the functional importance of the N-terminal interactions with the large lobe of the kinase domain that were observed *in silico*, we generated two constructs that delete the first 4 (ΔN4) or 8 residues (ΔN8). The two truncated enzymes exhibited 60–70% reductions in catalytic activity compared to the wild-type enzyme indicating that the unstructured N-terminal residues are, indeed, involved in an SH3-dependent activation of Csk. An additional construct was generated (D34A/ΔN13) that included a 13-residue N-terminal deletion and the individual D34A substitution. This construct eliminates the three charged contacts at the interface discussed so far and importantly D34A/ΔN13 exhibited a 6-fold reduction in kinase activity-the lowest second to ΔSH3 (Fig 3). Overall, these findings support the hypothesis that the *in silico* contacts between the N-terminus and kinase domain are equally important as those identified in the X-ray structure in supporting kinase function. Also, it appears that both sets of interactions act in an additive manner to support Src phosphorylation.

Since Csk’s kinase activity is a function of substrate binding and turnover rate, we measured the steady-state kinetic parameters of the variants (Table 1). The data indicate that deleting the entire SH3 domain led to a large ~6-fold reduction in k_cat_ and a ~4-fold increase in the K_m_ for kdSrc. These results imply that the reduction in observed catalytic activity for ΔSH3 is the result of changes in both protein substrate binding and maximum turnover rate. In contrast, the D34A variant which eliminates the charged side chain at the predicted interface exclusively reduces k_cat_ by ~6-fold without affecting substrate binding. Removal of the N-terminal residues has a much smaller effect on k_cat_ but increases the substrate K_m_ by 3-fold. These findings indicate that the N-terminus plays an important role in substrate binding. It is also interesting to note that K_m_ (ATP) was largely unaffected (Table 1) in all tested variants signifying that the functional integrity of the small kinase lobe motifs were not compromised. Overall, the steady-state kinetic data indicate that the contact regions serve different functions with regard to Src binding and turnover. Whereas the contacts identified in the X-ray structure (e.g. D34-R215) enhance maximum substrate turnover, the contacts identified *in silico* (N-terminus) that are missing in the X-ray structure are important for substrate binding.

**Table 1:** Kinetic parameters for select SH3 domain variants.

	Km (ATP), μM	kcat (kdSrc), min^-1^	Km (kdSrc), μM	kcat/km (kdSrc)	relative k_cat_/km
**WT CSK**	44.2±5.6	33.1±1.8	5.6±0.8	5.91	1
**ΔN8**	37.3±7.2	26.9±3.3	14.7±3.6	1.83	0.31
**D34A**	63.9±10	5.1±1.4	3.6±2.5	1.42	0.24
**ΔSH3**	39.1±9.0	5.1±0.5	22.8±4.1	0.22	0.04
**E13A**	ND	3.0±0.28	6.8±1.6	0.44	0.07
**D34A/ΔN13**	ND	37.7±4.3	23.4±4.5	1.61	0.27

Relative Steady-State kinetic values are listed for Csk variants and show differential effects on turnover rates, substrate binding, and catalytic efficiency.

### DXMS Studies Highlight Structural Conduits of Interdomain Communication

Structural and dynamic studies of Csk have revealed the importance of maintaining optimal dynamics for efficient catalysis [26–28]. Perturbations to large global domain motions are likely to shift the exchange between solution state ensembles that can be detected via established solution methods [29]. We sought to understand how the N-terminal (ΔN8) and SH3 domain truncations (ΔSH3) may influence the structural flexibility of the overall protein in solution. To address this, we evaluated the native dynamics of Csk using Deuterium Exchange Mass Spectrometry (DXMS) [30,31].

**Fig 4:**
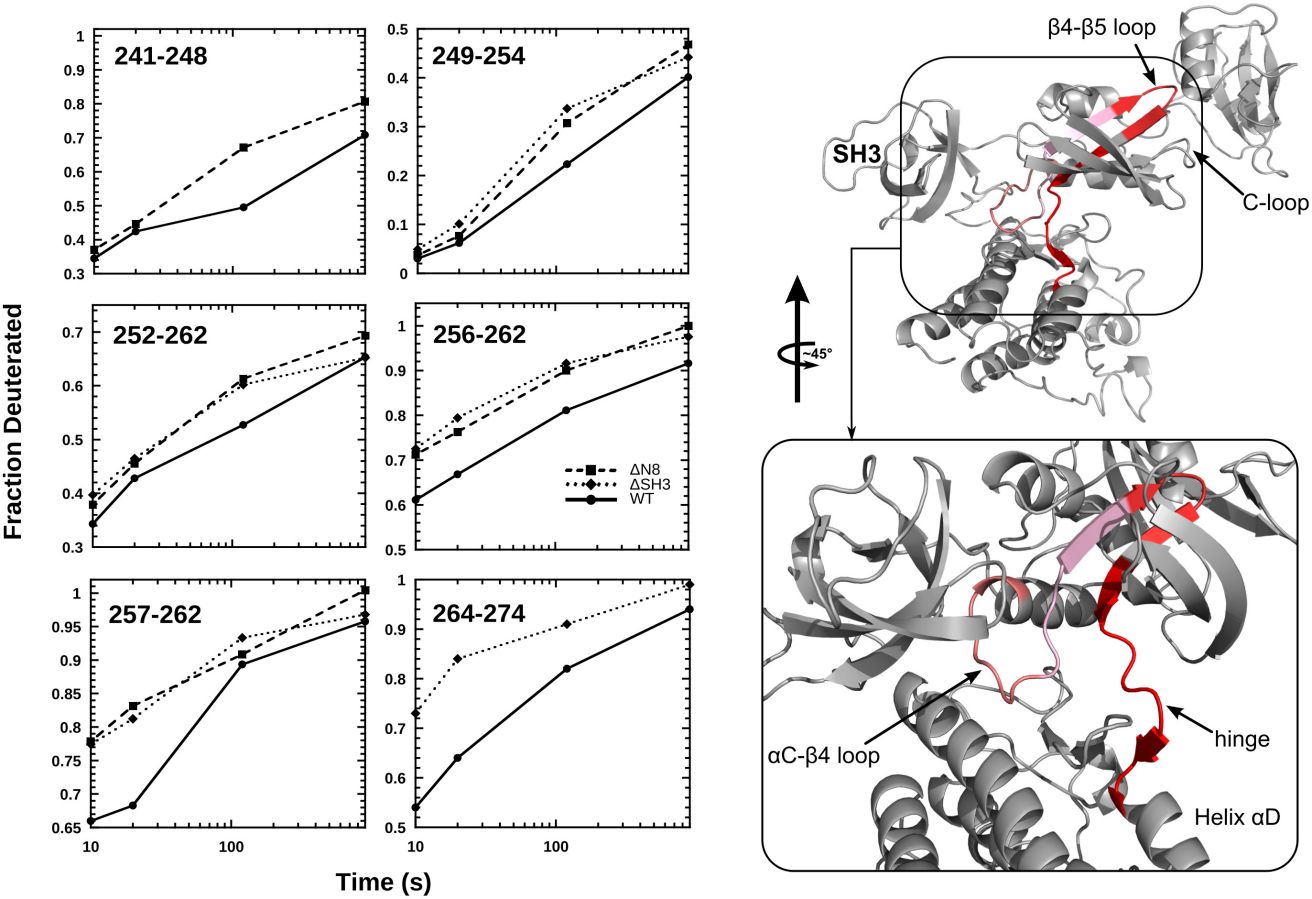
Effects of SH3 domain truncations on the time-dependent solvent deuterium incorporation into Csk peptide probes. Left: Deuterium incorporation into several probes in wild type, ΔN8, and ΔSH3 Csk is plotted as a function of time. Data were obtained over time courses of 1000 s at room temperature in deuterated buffer. Peptide identification and analysis were performed on two sets of data for verification. Right: Peptides with significant DXMS changes are plotted on the crystal structure of Csk. Red color gradient indicates level of HDX deprotection for each probe in the truncated constructs (ΔN8, ΔSH3) with respect to that observed for the wild type protein probes.

Select DXMS deuteration profiles are plotted for the two truncation variants in (Fig 4). Compared to wild-type Csk, both variants show higher uptake of solvent deuterons as a function of incubation time in a D_2_O buffer. This suggests that both the short and longer truncations perturb the same interaction mode, namely the SH3-dependent activation of the kinase domain. This deprotection effect is mostly localized to the core of the N-lobe of the kinase domain yet spans motifs that spatially bridge multiple functional ends of the molecule. A significant increase in solvent deuterium incorporation is detected for overlapping peptide probes that make up core motifs within the N-lobe such as the αC-β4 loop, β4-β5 loop, a region identified by Sun and coworkers as a critical network hub for interactions that stabilize and facilitate the active state of the N-lobe of the kinase domain in Csk [9]. Changes in solvent deuterium uptake are also localized to the kinase hinge region that extends to the start of αD—a motif that harbors important substrate binding residues (R271, R281) [32,33].

## Discussion

Over the past 10 years, many studies have established the presence of built-in mechanisms of kinase activation and inhibition that rely on peripheral motifs and modules. For SFKs, the tyrosine kinase domain possesses high, intrinsic catalytic activity that is down-regulated by interactions between the SH2 domain and a phosphorylated C-tail [34,35]. This inhibitory conformation can be relieved by a protein phosphatase that removes the C-tail phosphate or by an adapter protein such as Cbp/PAG that disrupts the inhibitory SH2-kinase interaction [34]. The importance of this inhibitory mode is evident in several oncogenic forms of c-Src that through mutation dislodge the inhibitory SH2 domain and present a high activity kinase domain that hyper-phosphorylates substrates important for cell division [36,37]. For Csk, the SH2 domain is not involved in such catalytic repression but instead takes on an activating role [10]. These large differences in regulatory mechanism are manifest in very different three-dimensional structures for the two kinases. Whereas the SH2 and SH3 domains interact with both the N- and C-lobes of the kinase domain in SFKs, they interact with only the N-lobe of the kinase domain of Csk [13]. These structural comparisons have led to an interesting hypothesis regarding how the weakly catalytic kinase domain of Csk is activated. For the kinase domain of c-Src, internal contacts within the N-lobe (R264 & C-loop) stabilize a hub centered on the β4–5 loop [9]. However, Csk appears to lack these internal contacts and instead relies on direct inter-domain contacts between a helix in the neighboring SH2 domain and the β4–5 loop. Although these contacts provide a rudimentary explanation for understanding how the SH2 domain activates the Csk kinase domain, they do not adequately provide a framework for understanding the role of the SH3 domain in enhancing kinase domain function.

In recent years it has become apparent that a complete picture of Csk regulation through domain contacts will not be fully realized through only a consideration of the X-ray structure of this kinase. Although the present structure offers several novel inter-domain contacts, other studies now reveal that the kinase adopts numerous forms in solution, some of which depart boldly from the conformation of the crystal structure. For example, recent SAXS studies from our lab demonstrate that Csk accesses an ensemble of structures in solution with many forms highly extended relative to the more compact X-ray structure [21,38]. These and other solution studies point to a highly dynamic structure where domains are likely to interact in unanticipated manners. In our aMD studies we show that the SH2 and SH3 domains in Csk can move in a synchronous fashion along the N-lobe of the kinase domain. We found that a high frequency motion in the SH2 correlates to large, transitory movement of the SH3 domain. We have characterized the nature of these movements and identified several contacts that appear to be important with regard to the SH3 domain movement. These contacts consist of two electrostatic pairs also visible in the X-ray structure along with several new contacts between the N-terminus of the SH3 domain and the C-lobe of the kinase domain. We demonstrate that these contacts detected both in the X-ray structure and *in silico* have significant effects on Src phosphorylation upon mutation. Notably, the kinetic studies indicate that contacts we identified *in silico* (N-terminus) that are missing in the X-ray structure are important for substrate binding.

**Fig 5:**
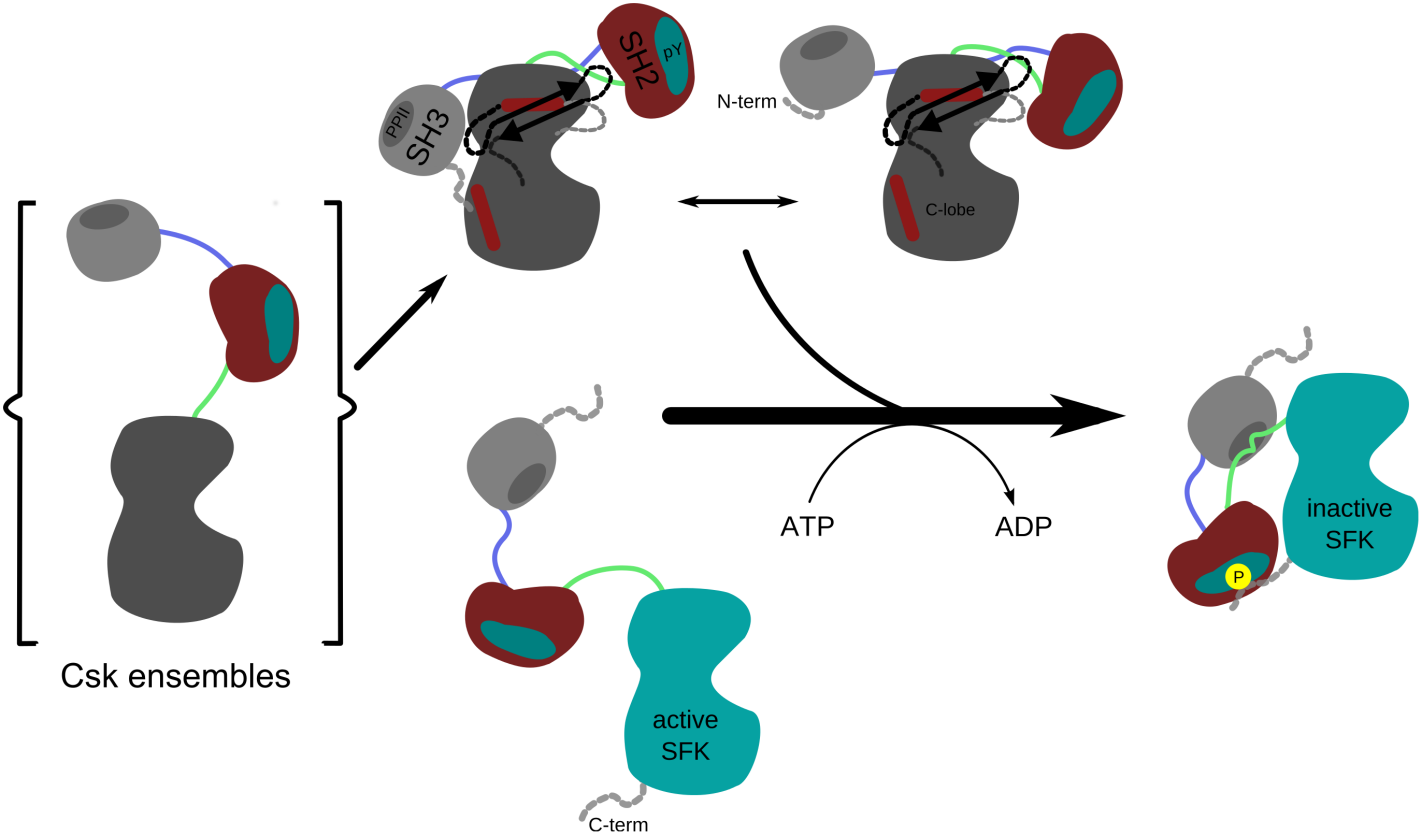
Dynamic interdomain activation in Csk. In the compact activated form, the functional motions of Csk’s modular domains are correlated for efficient activation of the kinase core to phosphorylate and downregulate SFKs.

The computational methods presented herein point to a new way to view the activation mechanism of Csk. Rather than relying solely on the static contacts visible in the X-ray structure, the aMD analyses expand upon these, define novel contacts and demonstrate that SH2 and SH3 domains move in opposing, yet seemingly coupled transitions. These motions are relayed bidirectionally where movement in one domain (SH2) is countered by a reversed one in another (SH3) with structural conduits traversing the kinase N-lobe facilitating these counter movements. Interestingly, we showed using DXMS that the hub identified in the N-lobe (β4–5 loop) is affected by these motions suggesting that the back and forth rocking of the SH2 and SH3 domains help to organize this critical region. This model is supported by the kinetic analyses which demonstrate that the disruption of contacts between the SH3 and kinase domain also lower Src phosphorylation rates. In prior studies we showed that Src phosphorylation is limited by conformational changes in Csk [27]. The model supported by this study (Fig 5) proposes that synchronous domain-domain motions of the type revealed in the aMD studies may underlie the rate-limiting conformational changes that limit Src phosphorylation and downregulation by Csk [27]. Overall, the variances among kinases can be dramatic despite most sharing canonical folds and modules. It appears that the dynamic modes of regulation that are now being elucidated in many signaling systems are key to understanding functional regulation [21,39,40]. As enhanced sampling methods become more desirable for biomolecular simulation studies (especially for groups with limited computational resources), the simulation-guided approach utilized here expands the applicability of aMD [23–25,41] to explore functional motions in systems with varying complexities ranging from small peptides to large membrane receptors. By studying a critical signaling kinase that regulates all SFKs, our results demonstrate that recent advancements in computational tools are a great asset in uncovering hidden functional motions in modular proteins that may be harder to assign when studied by classical biophysical techniques.

## Materials and Methods

### Protein Expression and Purification

(His)_6_ tagged Csk and kdSrc were expressed and purified as previously described [28]. Site-Directed mutagenesis was performed according to standard protocols. Deletion constructs were generated using the Phusion Site-Directed Mutagenesis Kit (Thermo Scientific). Enzymes were purified to near homogeneity as judged by SDS-PAGE analysis. A final purification step was performed on HiTrap Q HP anion exchange column (GE Healthcare) when deemed necessary.

### Kinase Activity Measurements

Time-dependent phosphorylation of kinase dead Src (kdSrc) by wild type or variant Csk enzymes was carried out in assay buffer: 100 mM MOPS (pH 7.0), 100 mM NaCl, 10 mM MgCl_2_, 1mM MnCl_2_, 100 μM [γ-^32^P]ATP (4000–6000 cpm pmol^-1^) and 10 mM DTT at lab temperature (23°C). Reactions were initiated by adding ATP to the master mix containing all enzymes and reagents. a fraction of the reaction was quenched at several time intervals and subjected to SDS-PAGE. Bands corresponding to the phosphorylated kdSrc substrate were excised and quantified on the ^32^P channel in liquid scintillator. The specific activity of [γ-^32^P]ATP was determined by measuring the total counts of the reaction mixture. The time-dependent concentration of ^32^P-kdSrc was then determined by considering the total counts per minute (CPM), the specific activity of the reaction mixture, and the background phosphorylation. Non-linear regression analysis were performed to quantify the activity of each construct from phosphorylation time course of a fixed amount of kdSrc. The data were fitted to the following equation to obtain rates (half-life): P = A(1-e^kt^) where P is the phospho-product, A is the total substrate concentration, k is the rate constant, and t is time. Initial velocity experiments were typically initiated with variable amounts of substrates (kdSrc or ATP) and data were fitted to a Michaelis–Menten model to obtain steady state parameters.

### Deuterium Exchange-Mass Spectrometry (DXMS)

Optimal proteolysis conditions for CSK were previously established [28].

#### DXMS Operation

The instrument setup and operation was previously described [42]. All frozen samples were thawed and run using the conditions determined during fragmentation optimization.

#### Deuterium on-exchange Experiments

The exchange time course experiments for wild type and variant Csk samples were all performed simultaneously at 23°C as follows: Deuteration was initiated by adding 24 μL of ~20 μM protein stock solution (50 mM Tris, 150 mM NaCl, 10% glycerol, 2 mM DTT, pH 7.0) to 120 μL of the equivalent deuterated exchange buffer for a final D_2_O of 83%. The deuterated exchange buffer (50 mM d-Tris, 150 mM NaCl, 10% glycerol, 2 mM d-DTT) was prepared using 99.9% D_2_O and adjusted to pD 7.0 with DCl. 24 μL of the exchange reaction was quenched at specified time points (10, 20, 120, 900 s) into pre-chilled high recovery autosampler vials containing 6 μL of quench buffer (2.5M Gdn-HCl, 225mM TCEP, 2.5% Formic acid, 25% Glycerol). The vials were sealed and frozen over dry ice, then stored at -80°C until analysis. The in-exchange control consisted of the protein added directly to the pre-chilled deuterated and quench buffers, then immediately followed by the normal sample preparation procedure. The back exchange control was performed by incubating samples in 0.5% formic acid in D_2_O buffer overnight.

#### Sequence Identification of Peptide Fragments

The identity of the parent peptide ions was determined using the Proteome Discoverer software program (ThermoFisher) and data dependent MS/MS data. The quality of each peptide was monitored by individually examining each measured isotopic envelope spectrum for the entire time course exchange. The deuterium content was calculated for each time point by using specialized software as previously described [43].

### Computational Methods

The PDB structure 1K9A was prepared using Schrodinger’s protein preparation wizard, modeling in residues of the N-terminus and the activation loop which are not observed in the X-ray structure. The structure was minimized and solvated in an octahedral TIP3P water box. The system was then equilibrated and a 50 ns conventional molecular dynamics NVT simulations was run using the AMBER ff99SB-ILDN force field [44]. The standard aMD protocol was used [41]; average dihedral and total potential energies were calculated from the 50 ns cMD simulation and used to select the acceleration parameters used in the 500 ns accelerated molecular dynamics simulation (aMD).

## Supporting Information

S1 Fig Correlated motioned observed for the SH2 and SH3 domains in Csk. The root mean square deviations (RMSD) for the SH3 domain (blue, residues 8–68) and SH2 domain (orange, residues 80–125) are plotted as a function of aMD simulation time. The calculated values are relative to the kinase domain throughout the simulation time.
(TIF)

S2 Fig SH3 domain transitions. Computed RMSD of the SH3 domain relative to the crystal structure of CSK for the long trajectory (A) and independent 175 ns simulations (sims B-F). The same transition seen in the long simulation is also observed in shorter simulation (E).
(TIFF)

S3 Fig Comparison of contacts between two states from two independent simulations. Frames extracted from two independent simulations are clustered into two states based on the RMSD of the SH3 domain. The fractional occupancy of contacts present in the two states are compared for two simulations (A: top; and E: bottom) in which the SH3 domain is designated as "up, state 0” or "down, state 1”. Similar occupancy changes are observed for many contacts between the two states indicating that both simulations report on the same transition of the SH3 domain.
(TIF)

S1 File aMD simulations of Csk. Frames extracted from the long and short aMD trajectories are used to visualize the motions of regulatory domains in Csk. Similar motions can be observed in the the long (500 ns, blue) and the shorter (175 ns, others) aMD simulations.
(MPG)
