# Correction: Theoretical Insights Reveal Novel Motions in Csk’s SH3 Domain That Control Kinase Activation

**DOI:** 10.1371/journal.pone.0133801

**Published:** 2015-07-20

**Authors:** Sulyman Barkho, Levi C. T. Pierce, Sheng Li, Joseph A. Adams, Patricia A. Jennings

There is information missing from funding section of this paper. JAA is supported by NIH grants GM067969 and GM098528.
