# Supplementary figures and images for: Theoretical Insights Reveal Novel Motions in Csk’s SH3 Domain That Control Kinase Activation
Source: PLoS One. 2015 Jun 1;10(6):e0127724. doi: 10.1371/journal.pone.0127724 (PMC4452171; doi:10.1371/journal.pone.0127724)

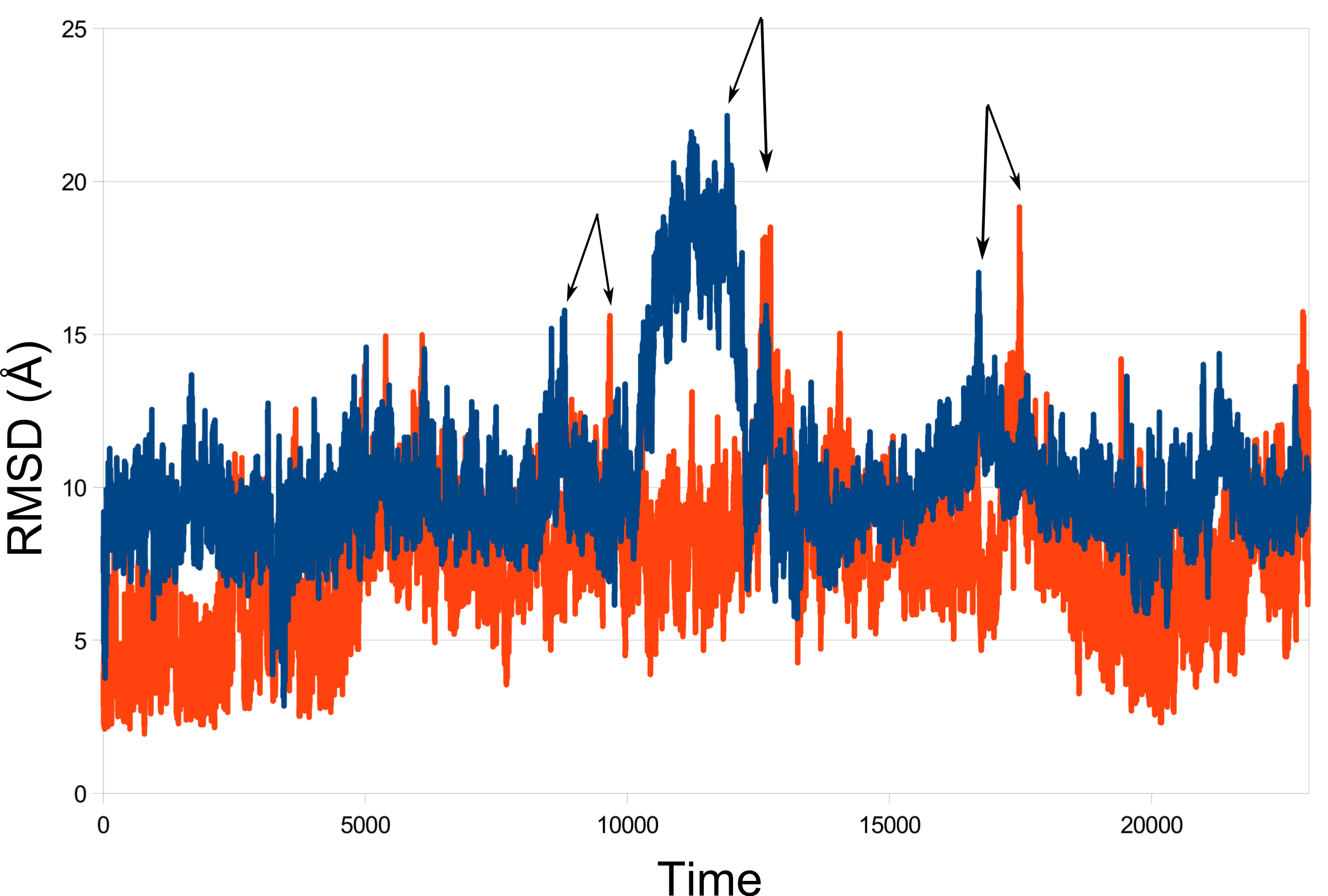

Supplement: S1 Fig — The root mean square deviations (RMSD) for the SH3 domain (blue, residues 8–68) and SH2 domain (orange, residues 80–125) are plotted as a function of aMD simulation time. The calculated values are relative to the kinase domain throughout the simulation time. (TIF) [file pone.0127724.s001.tif]

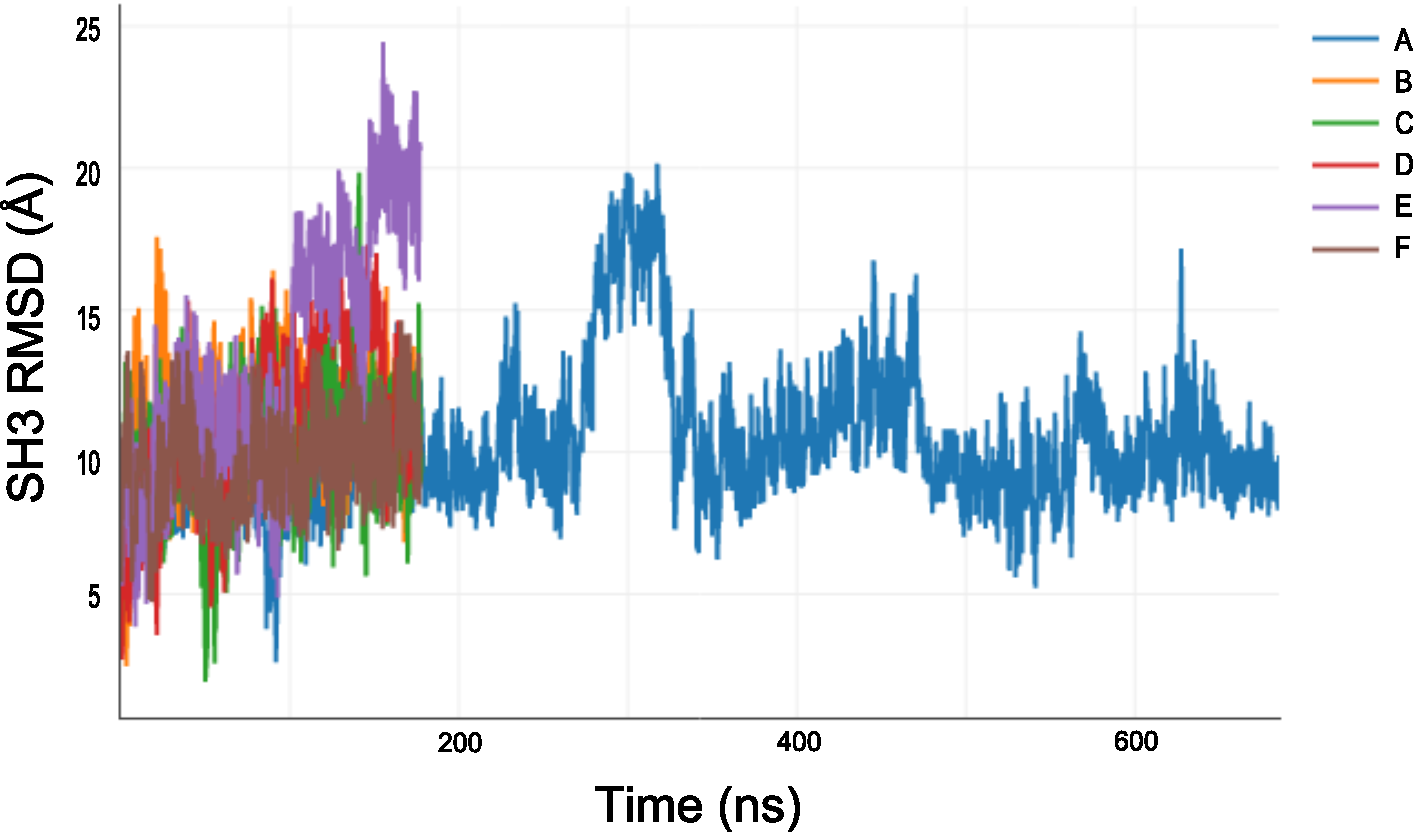

Supplement: S2 Fig — Computed RMSD of the SH3 domain relative to the crystal structure of CSK for the long trajectory (A) and independent 175 ns simulations (sims B-F). The same transition seen in the long simulation is also observed in shorter simulation (E). (TIFF) [file pone.0127724.s002.tiff]

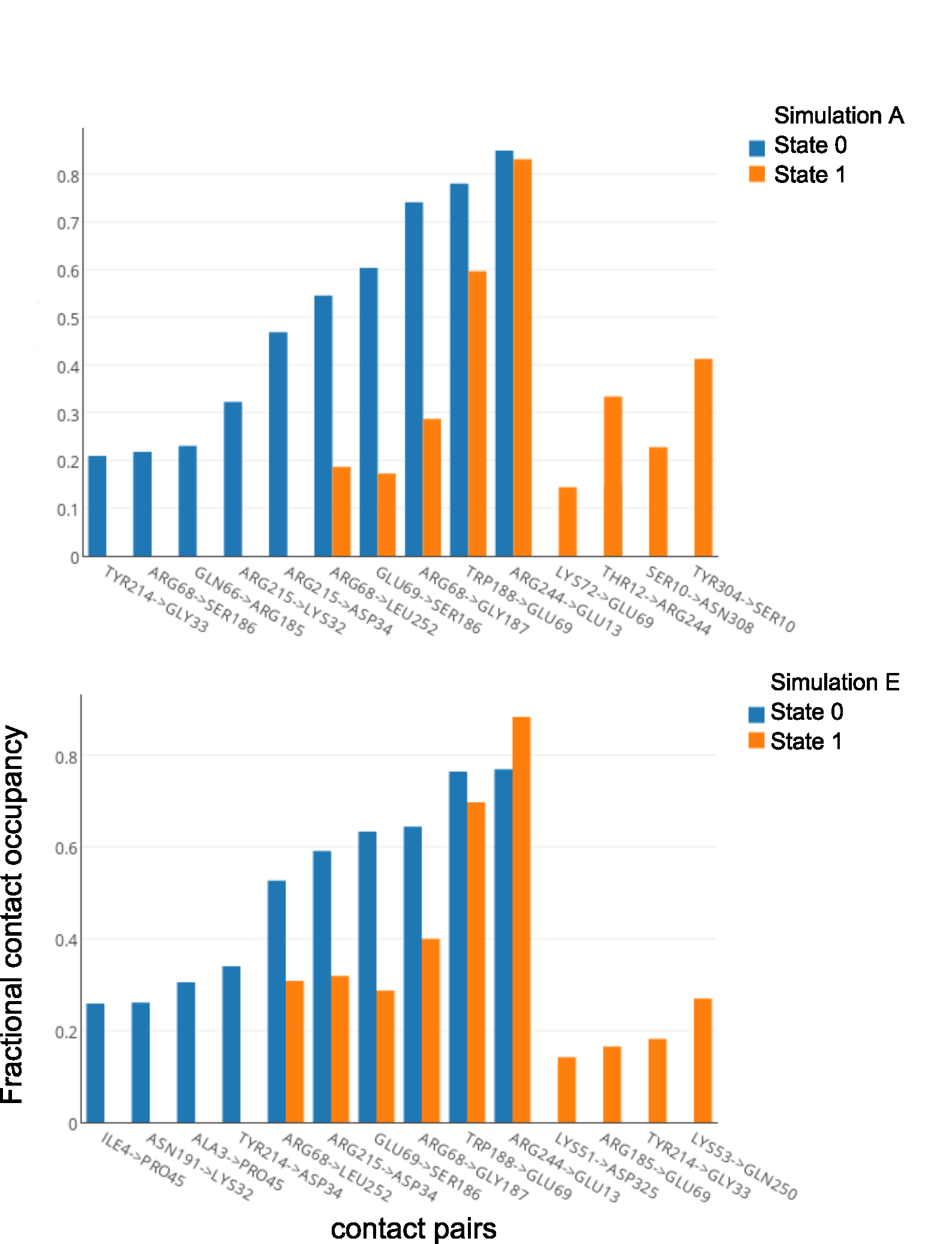

Supplement: S3 Fig — Frames extracted from two independent simulations are clustered into two states based on the RMSD of the SH3 domain. The fractional occupancy of contacts present in the two states are compared for two simulations (A: top; and E: bottom) in which the SH3 domain is designated as "up, state 0” or "down, state 1”. Similar occupancy changes are observed for many contacts between the two states indicating that both simulations report on the same transition of the SH3 domain. (TIF) [file pone.0127724.s003.tif]
